# Impact of delayed cord clamping on mesenchymal, hemopoietic progenitor, and immune cells in very preterm neonates

**DOI:** 10.3389/fped.2025.1698512

**Published:** 2025-11-25

**Authors:** Eftychia Drogouti, Dimitrios Rallis, Alexandra Fleva, Anastasia Giannakou, Maria Lithoxopoulou, Maria Kavga, Themistoklis Mikos, Vasiliki Soubasi, Elisavet Diamanti, Emmanuel Roilides, Christos Tsakalidis

**Affiliations:** 1 Second Department of Neonatology, School of Medicine, Aristotle University of Thessaloniki, Thessaloniki, Greece; 2 Department of Immunology, Papageorgiou General Hospital, Thessaloniki, Greece; 3 Third Department of Pediatrics, School of Medicine, Aristotle University of Thessaloniki, Thessaloniki, Greece; 4 Unit of Reproductive Endocrinology, First Department of Obstetrics and Gynecology, School of Medicine, Aristotle University of Thessaloniki, Thessaloniki, Greece

**Keywords:** immunophenotype, neonates, umbilical cord, late onset sepsis, bronchopulmonary dysplasia

## Abstract

**Objective:**

We aimed to investigate the impact of delayed cord clamping (DCC) on the levels of blood mesenchymal (MSCs), hemopoietic progenitor (HPCs), and immune cells in very preterm neonates.

**Methods:**

We prospectively examined 21 neonates with a median gestational age of 32 weeks (interquartile range, IQR 29–32) who had DCC, and 19 neonates with a median gestational age of 31 weeks (IQR 30–32) who had immediate cord clamping (ICC). We measured the levels of MSCs, HPCs, very small embryonic-like stem cells (VSELs), early endothelial progenitor cells (EPCs), late EPCs, and the immunophenotype in the first, 10th, and 30th day of life, and associated them with late-onset sepsis and bronchopulmonary dysplasia (BPD).

**Results:**

DCC-neonates compared to ICC-neonates had significantly higher values of MSCs (846 vs. 316 cells per million cytometric events, *p* = 0.003), HPCs (191 vs. 115 cells per million cytometric events, *p* = 0.034), and lower values of VSELs (21 vs. 37 cells per million cytometric events, *p* = 0.044) and late EPCs (7 vs. 19 cells per million cytometric events, *p* = 0.017) at birth. Neonates with late-onset sepsis, in comparison to neonates with no sepsis, had significantly higher values of early (20 vs. 0.3 cells per million cytometric events, *p* = 0.011) and late EPCs (32 vs. 8 cells per million cytometric events, *p* = 0.033). In addition, neonates with BPD had significantly higher values of late EPCs compared to neonates without BPD (27 vs. 8 cells per million cytometric events, *p* = 0.041). DCC, adjusted for gestational age and birth weight, was significantly associated with higher levels of MSCs, HPCs, and lower levels of VSELs and late EPCs.

**Conclusions:**

In very preterm neonates with DCC, MSCs and HPCs are higher, while VSELs and late EPCs are lower in the umbilical cord blood, compared to neonates with ICC. Early and late EPCs were associated with late-onset sepsis and BPD. Further studies are warranted to explore the association of these findings with the long-term clinical outcomes.

## Introduction

There has been much discussion over the years regarding when to clamp the umbilical cord (1). Currently, umbilical cord clamping should be deferred for at least 60 s in preterm neonates born at less than 37 weeks of gestation who do not require immediate resuscitation at birth (2). Numerous studies have demonstrated the advantages of delayed umbilical cord clamping (DCC), which include decreased risks of intraventricular hemorrhage, necrotizing enterocolitis, elevated hemoglobin concentration, and enhanced iron reserves (3, 4). Furthermore, umbilical cord blood (UCB) is a desirable source of stem cells because it is painless, noninvasive, and does not raise the same ethical issues as embryonic stem cell transfusion (5). Since cord blood volume and stem cell production are strongly correlated, DCC has been carefully examined because of its potential to reduce the amount of blood that remains in the placenta and UCB (5).

Several studies have suggested that DCC might be the best noninvasive method for delivering autologous stem cells (1, 6). Approximately 33% of the feto-placental blood volume remains in the placenta after immediate cord clamping (ICC), whereas 20% of the feto-placental blood volume remains at DCC after 60 s and 13% after 3 min (7). A variety of stem and progenitor cell types, such as mesenchymal stem cells (MSCs), hemopoietic progenitor cells (HPCs), endothelial progenitor cells (EPCs), endothelial colony-forming cells (ECFCs), a subset of EPCs with endothelial appearance, highly proliferative, self-renewing, and angiogenic properties (8), and very small embryonic-like stem cells (VSELs) are found in UCB (9, 10); thus, UCB is an important source of stem and progenitor cells (11). Because the cord blood of very preterm neonates (i.e., those who are born below 32 weeks of gestational age) contains more HPCs and long-term culture-initiating cells than that of term neonates, the postnatal transfer of UCB may be especially crucial in these cases (12). The findings of previous research have been conflicting; some studies have suggested that DCC is associated with higher quantities of MSCs and EPCs (13, 14), while other studies have found that neonates with DCC had lower amounts of stem cells than their ICC counterparts (15). Furthermore, there is still little data linking MSCs and primitive HPCs to common neonatal outcomes like late-onset sepsis (i.e., sepsis that occurs after the first 72 h of life) and bronchopulmonary dysplasia (BPD). Some authors have found an association between lower levels of EPCs, or ECFCs, and sepsis or BPD (16–19), while others have found higher levels of EPCs in neonates with sepsis or BPD (20), and still others have found no correlation at all (21, 22).

Given the above evidence, our study aimed to investigate the impact of DCC on the levels of blood MSCs, HPCs, VSELs, early and late EPCs, and immunophenotype, and the association of primitive stem cells with neonatal outcomes such as sepsis and BPD in very preterm neonates.

## Materials and methods

A prospective cohort study was conducted at the Second Neonatal Unit of the Aristotle University of Thessaloniki, Papageorgiou Hospital, Greece, from December 2018 to July 2022, enrolling 40 preterm neonates of ≤32 weeks of gestational age. An analysis was performed between neonates with DCC compared to neonates with ICC. The data were pseudonymized, and the study was approved by the Ethical Committee of the Aristotle University of Thessaloniki (No 3/2.5.18). All parents signed an informed consent prior to participating in the study.

All neonates who were born between 22 and 32 weeks of gestational age, according to the last menstrual period and/or early pregnancy ultrasound, were eligible for inclusion in the study. When there was discrepancy between the last menstrual period and the early pregnancy ultrasound, gestational age was estimated based on the early pregnancy ultrasound. In our institution, according to the latest American College of Obstetricians and Gynecologists (ACOG) recommendations (2), DCC was attempted in all neonates and was only omitted in cases with contraindications. In case that DCC was contraindicated (i.e., neonates requiring resuscitation at birth, neonates with perinatal asphyxia, or neonates with placental abruption), then immediate cord clamping (ICC), i.e., cord clamping at less than 10 sec, was performed. Neonates who were born with any of the following conditions were excluded from the study: (1) chromosomal anomaly, (2) congenital dysplasia (both structural malformations and genetic syndromes), or (3) maternal chorioamnionitis defined by the ACOG criteria (23). The perinatal and neonatal characteristics that were recorded included gestational age, birth weight, sex, mode of delivery, small for gestational age (defined by a birth weight below the 10th centile according to the Intergrowth charts), pregnancy-induced hypertension, maternal gestational diabetes, multiparity, mode of conception, complete course of antenatal steroid administration, prolonged rupture of membranes, Apgar scores, respiratory distress syndrome manifestation, days on invasive and non-invasive ventilation, patent ductus arteriosus, early- and late-onset sepsis, number of red blood cell transfusions, necrotizing enterocolitis, intraventricular hemorrhage, retinopathy of prematurity, BPD, and the length of hospital stay. Neonatal outcomes were defined according to the Vermont-Oxford Network criteria (24), while BPD was defined according to the Jensen criteria (25).

The blood gas analyses, full blood count, C-reactive protein, the percentages of basic immunophenotype of lymphocytes, MSCs, HPCs, VSELs, and EPCs were measured in the umbilical cord of every neonate in the study. Peripheral blood samples were also drawn from each neonate on the 10th day of life, and another one on the 30th day of life for subsequent measurements of the same parameters. Briefly, after cord clamping, an umbilical blood sample of 1 ml was collected in an EDTA tube and brought to the hematology laboratory. White blood cell counts were measured using the Cell-Dyn sapphire (Abbott Laboratories, IL, USA) automated cell counter. One sample without coagulant was taken to the biochemistry laboratory.

**Figure 1:**
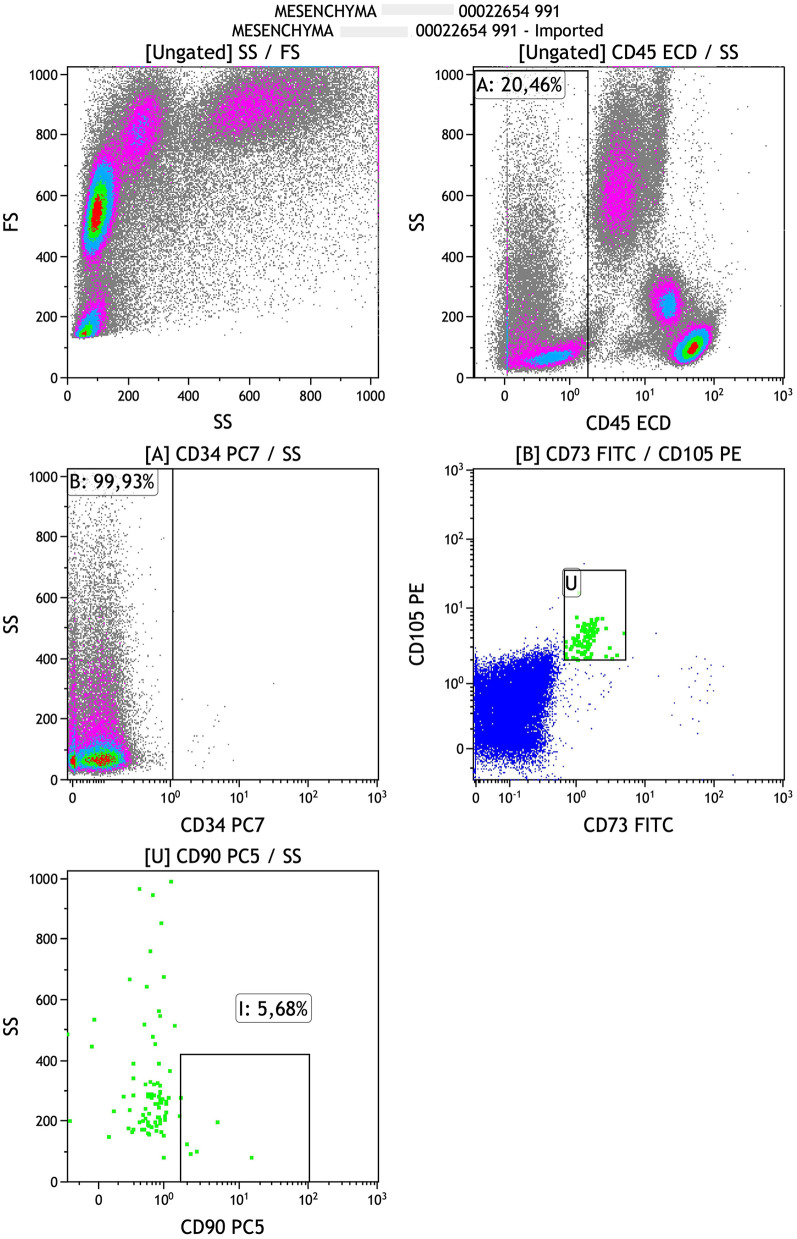
Gating strategy of mesenchymal cells.

**Figure 2:**
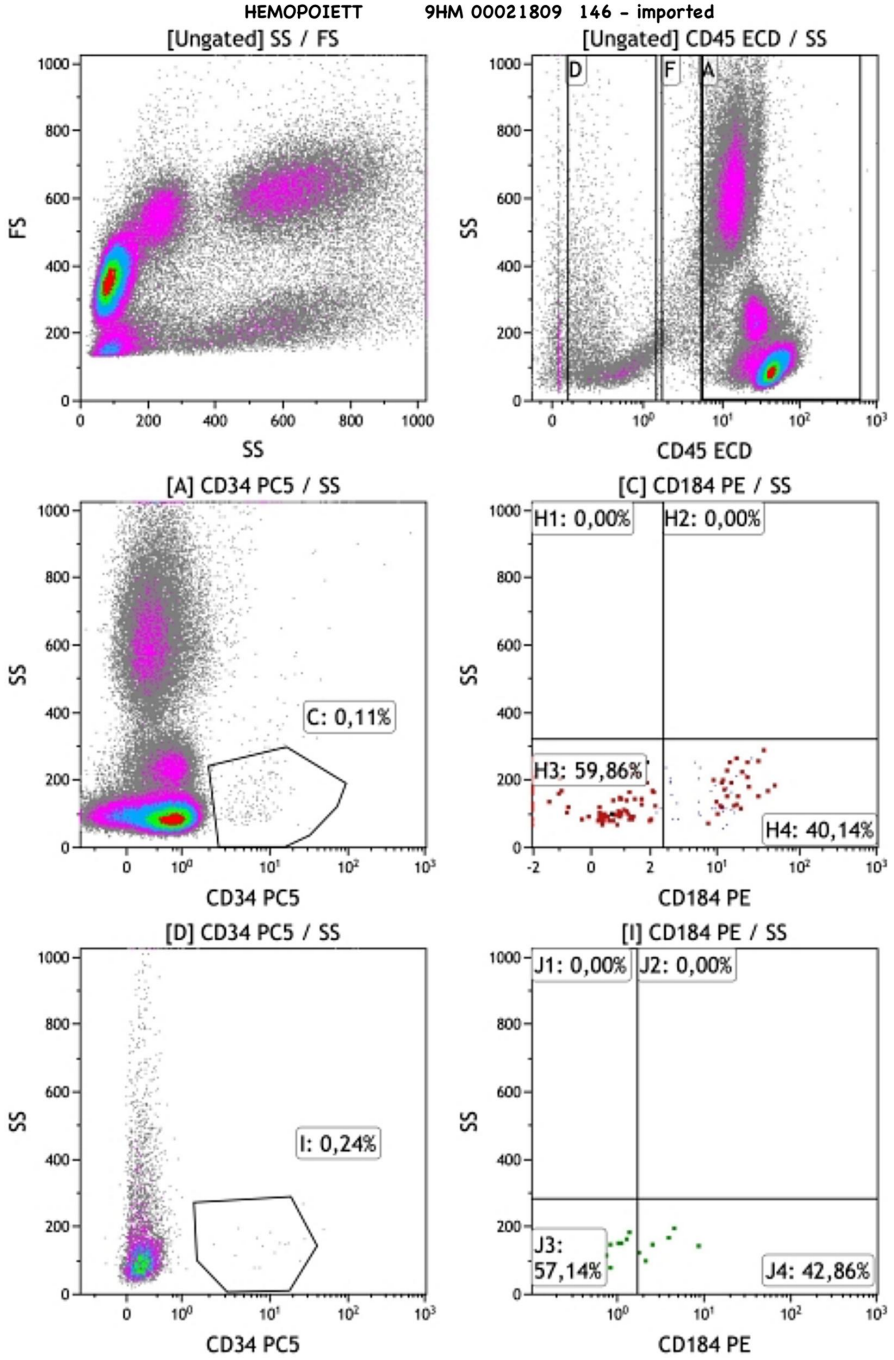
Gating strategy of hemopoietic progenitor cells.

Finally another anticoagulated umbilical cord sample was taken to the Flow Cytometry Laboratory where the percentages of MSCs (CD45−/CD34−/CD73+/CD90+/CD105+), HPCs (CD184 + PE/CD34 + PC5/CD45 + ECD), VSELs (CD184 + PE/CD34 + PC5/CD45−ECD), early EPCs (CD133 + FITC/CD184 + PE/CD34PC5/CD45dim/-ECD), late EPCs (CD133-FITC/CD184 + PE/CD34 + PC5/CD45 dim/-ECD), and the immunophenotype of T,B and NK cells (CD3 + FITC, CD3 + FITCCD4 + PE, CD3 + FITCCD8 + PE, CD19 + PE, CD3-FITCCD16 + 56 + PE) were measured by five-color flow cytometry using the FC 500 flow cytometer (Beckman Coulter, USA) and monoclonal antibodies by Beckman Coulter and EXBIO (Supplementary Table 1). Cells were gated by singlets followed by forward vs side scatter based on size and complexity. No viability testing was performed. It should be noted that EPCs although a heterogeneous population, were identified as CD133 + cells only. This could be proven to be a limitation for the study of these cells. For VSLs, recognizing that it is a rare stem cell population, we decided to base our identification on CD184+ (CXCR4) pluripotency marker and CD45 negative CD34 positive lineage. A representative analysis of the gating strategy of MSCs and HPCs is presented in Figures 1, 2, respectively.

### Statistical analysis

The primary outcome of the study was to examine the impact of DCC compared to ICC on the levels of the MSCs, HPCs, VSELs, early EPCs, late EPCs, and the immunophenotype in very preterm neonates. Secondary outcomes were the evaluation of the levels of the same parameters in neonates with and without late-onset sepsis and in neonates with and without BPD.

Continuous variables were expressed as median (interquartile range, IQR). The normality of the distributions of continuous variables was assessed by the Shapiro–Wilk test. Comparisons of continuous variables between the DCC and ICC groups, as well as between neonates with and without late-onset sepsis, and neonates with and without BPD were performed utilizing the non-parametric Mann–Whitney test. Categorical variables were expressed as n (percentage %) and compared between the DCC and ICC groups with the chi-square test or Fisher's exact test. Since the MSCs, HPCs, VSELs, early EPCs, late EPCs, and the immunophenotype were not normally distributed, the comparisons of those parameters within each group between the first, 10th, and 30th day of life were performed with the Friedman test with *post hoc* analysis with the Bonferroni correction.

A multivariable linear regression analysis was used to examine the association of DCC with MSCs, HPCs, VSELs, early EPCs, and late EPCs at birth, adjusted for gestational age and birth weight. The Bonferroni correction for multiple testing was utilized, while the coefficient b and the confidence intervals (CI) were calculated. All tests were two-sided, and a p-value less than 0.05 was considered statistically significant (alpha 0.05). The data were analyzed using SPSS Statistics (IBM SPSS Statistics for Windows, Version 26.0. Armonk, NY, USA).

## Results

During the study period, we included 40 very preterm neonates with a median gestational age of 31 (IQR 29–32) weeks and a median birth weight of 1,255 (IQR 1,239–1,450) g. Among them, 19 (48%) neonates were males, and 29 (73%) were born by cesarean section. Overall, 32 (80%) neonates developed RDS, and 6 (15%) neonates developed BPD. Early-onset sepsis was not recorded in any neonate, while late-onset sepsis was recorded in 6 (15%) neonates (Table 1).

**Table 1:** Perinatal characteristics of the neonatal cohort, and between neonates with DCC and ICC.

Perinatal characteristics	Total cohort (*n* = 40)	DCC (*n* = 21)	ICC (*n* = 19)	*p*
Gestational age, weeks	31 (29–32)	32 (29–32)	31 (30–32)	0.320
Birth weight, g	1255 (1,239–1,450)	1,250 (1,244–1,392)	1,320 (1,180–1,590)	0.251
Sex, male	19 (48%)	11 (52%)	8 (42%)	0.545
Mode of delivery, cesarean section	29 (73%)	16 (76%)	13 (68%)	0.727
Small for gestational age	8 (20%)	3 (14%)	5 (26%)	0.442
Pregnancy-induced hypertension	1 (3%)	–	1 (5%)	0.475
Maternal gestational diabetes	3 (8%)	2 (9%)	1 (5%)	1.000
Multiparity	10 (25%)	4 (19%)	6 (31%)	0.473
Placental abruption	11 (28%)	–	11 (58%)	0.004*
Conception, *in-vitro* fertilization	8 (20%)	5 (23%)	3 (16%)	0.698
Antenatal steroids	35 (88%)	19 (90%)	16 (84%)	0.654
Prolonged rupture of membranes	8 (20%)	3 (14%)	5 (26%)	0.442
Positive-pressure ventilation needed at the delivery room	8 (20%)	–	8 (42%)	0.005*
APGAR first minute	8 (7–8)^a^	8 (7–8)^a^	8 (7–8)^a^	0.592
APGAR fifth minute	8 (8–9)^a^	8 (8–9)^a^	8 (8–9)^a^	0.869

Continuous variables are expressed as median (interquartile range). *P*-value of the Mann–Whitney *U* test. Categorical variables are expressed as *n* (%). *P*-value of the chi-square test.

DCC, delayed cord clamping; ICC, immediate cord clamping.

* Statistically significant.

a Non-parametric variables.

Among the study cohort, DCC was performed in 21 neonates of 32 (IQR 29–32) weeks of gestational age and 1250 (IQR 1,244–1,392) g of birth weight, while ICC was performed in 19 neonates with a median gestational age of 31 (IQR 30–32) weeks and 1320 (IQR 1,180–1,590) g of birth weight (*p* = 0.320 and 0.251, respectively). Eleven neonates with ICC were born to mothers with placental abruption, while 9 neonates had ICC because they required positive-pressure ventilation at birth. No significant differences were recorded between neonates with DCC and ICC in the mode of delivery, mode of conception, pregnancy-induced hypertension, maternal diabetes, antenatal steroids administration, APGAR scores, or the neonatal outcomes, as shown in Tables 1, 2.

**Table 2:** Outcomes of the neonatal cohort, and between neonates with DCC and ICC.

Outcomes	Total cohort (*n* = 40)	DCC (*n* = 21)	ICC (*n* = 19)	*p*
Respiratory distress syndrome	32 (80%)	17 (81%)	15 (79%)	1.000
Mechanical ventilation duration, days	0 (0–0)^a^	0 (0–0)^a^	0 (0–1)^a^	0.630
Non-invasive ventilation duration, days	1 (0–5)^a^	1 (0–4)^a^	4 (0–9)^a^	0.296
Patent ductus arteriosus	3 (8%)	2 (9%)	1 (5%)	1.000
Early onset sepsis	–	–	–	n/a
Late onset sepsis	6 (15%)	3 (14%)	3 (15%)	1.000
Necrotizing enterocolitis	1 (3%)	1 (4%)	–	1.000
RBC transfusion, times	0 (0–0)^a^	0 (0–0)^a^	0 (0–0)^a^	1.000
Phototherapy, days	3 (2–4)^a^	3 (2–4)^a^	3 (2–4)^a^	0.837
Intraventricular hemorrhage	3 (8%)	2 (9%)	1 (5%)	1.000
Retinopathy of prematurity	–	–	–	n/a
Bronchopulmonary dysplasia	6 (15%)	2 (9%)	4 (21%)	0.398
Length of stay, days	25 (11–55)^a^	20 (10–55)^a^	25 (14–50)^a^	0.830
Survival	40 (100)	21 (100)	19 (100)	n/a

Continuous variables are expressed as median (interquartile range) as appropriate. *P*-value of the Mann–Whitney *U* test. Categorical variables are expressed as *n* (%). *P*-value of the chi-square test.

DCC, delayed cord clamping; ICC, immediate cord clamping; RBC, red blood cell.

a Non-parametric variables.

Neonates with DCC compared to those with ICC had significantly higher median values of MSCs (846 vs. 316 cells per million cytometric events, *p* = 0.003), HPCs (191 vs. 115 cells per million cytometric events, *p* = 0.034), and lower median values of VSELs (21 vs. 37 cells per million cytometric events, *p* = 0.044) and late EPCs (7 vs. 19 cells per million cytometric events, *p* = 0.017) at birth (Table 3). We recorded no significant differences between the two groups in any other parameters on the 10th or 30th day of life. In repeated comparisons of hematological parameters within the DCC and ICC groups, we found that MSCs were significantly reduced from birth to the 10th and 30th day of life, and HPCs were significantly reduced from birth to the 10th day of life in the neonates with DCC only (Table 3).

**Table 3:** Hematological parameters between neonates with DCC and ICC on the first, 10th and the 30th day of life.

Hematological parameters	Day 1			Day 10			Day 30		
	DCC (*n* = 21)	ICC (*n* = 19)	*p*	DCC (*n* = 21)	ICC (*n* = 19)	*p*	DCC (*n* = 21)	ICC (*n* = 19)	*p*
MSCs ^a^	846 (361–2282)	316 (182–419)	0.003*	299 (197–605)^c^	332 (146–573)	0.728	71 (35–201)^c^	262 (100–404)	0.143
HPCs ^a^	191 (105–389)	115 (59–174)	0.034*	117 (44–145)^c^	69 (39–133)	0.769	168 (58–248)	149 (66–197)	0.971
VSELs ^a^	21 (10–50)	37 (15–105)	0.044*	16 (9–38)	16 (8–29)	0.635	44 (14–108)	39 (22–69)	0.912
Early EPCs ^a^	0.3 (0.03–0.6)	1.9 (0.08–14)	0.977	2 (0.1–5)	3 (0.01–8)	0.946	1.5 (0.06–5)	4.3 (0.04–8)	0.684
Late EPCs ^a^	7 (0.3–22)	19 (11–33)	0.017*	8 (2–20)	6 (3–22)	1.000	8 (5–45)	18 (12–32)	0.481
White blood cells (cells ×10^3^)	12.7 (10.9–15.2)	10.6 (8.8–19.8)	0.602	14.4 (13.9–15.9)	11.6 (10.6–15.9)	0.221	12.9 (10.5–14.1)	9.9 (7.5–15.4)	0.683
Neutrophils, %	58 (53–66)	50 (41–62)	0.422	39 (29–45)	38 (25–44)	1.000	22 (11–36)	26 (14–38)	1.000
Lymphocytes, %	28 (22–37)	38 (28–41)	0.345	42 (42–59)	44 (36–56)	0.913	65 (44–75)	51 (28–61)	0.368
Hemoglobin, g/dl	16.8 (15.1–18.2)	15.9 (14.8–17.3)	0.554	15.1 (14.2–15.3)	14.3 (12.9–15.3)	0.377	11.6 (11.2–12.1)	9.7 (8.8–10.4)	0.048*
Hematocrit, %	49.4 (45.9–53.7)	48.2 (46.2–53.9)	0.862	46.2 (44.0–53.3)	42.9 (39.3–44.5)	0.090	34.6 (32.8–35.7)	30.4 (26.2–32.8)	0.154
Reticulocytes, %	5.9 (4.7–6.3)	5.7 (4.7–7.2)	0.917	1.5 (1.4–1.5)	1.8 (1.3–5.5)	0.377	2.7 (2.1–3.2)	2.2 (2.1–3.7)	0.368
Platelets (cells ×10^3^)	232 (231–255)	212 (184–241)	0.219	200 (150–401)	320 (254–441)	0.165	288 (240–481)	361 (320–495)	0.933
CRP, mg/dl	0.15 (0.12–0.20)	0.24 (0.17–0.47)	0.122	0.16 (0.14–0.85)	0.38 (0.18–1.10)	0.412	0.15 (0.13–0.16)	0.16 (0.15–0.19)	0.389
CD3 ^b^ ^,^ ^c^	73.2 (61.3–79.3)	77.5 (71.8–82.2)	0.111	78.8 (70.2–80.8)	79.1 (75.1–80.6)	0.464	74.6 (68.2–78.1)	65.5 (58.9–77.8)	0.393
CD3^+^/CD4 ^b^ ^,^ ^c^	49.6 (42.5–58.4)	59.2 (49.7–63.0)	0.034*	60.4 (51.1–61.8)	59.1 (54.1–62.7)	0.682	53.2 (52.1–60.1)	45.5 (42.6–55.3)	0.143
CD3^+^/8 ^b^ ^,^ ^c^	18.8 (15.8–27.1)	19.6 (16.9–22.4)	0.688	17.1 (14.8–20.7)	18.3 (14.5–21.5)	0.708	16.8 (15.6–19.2)	17.8 (13.4–22.5)	0.912
CD19 ^b^ ^,^ ^c^	12.8 (10.2–20.5)	10.1 (5.8–12.9)	0.029*	12.4 (8.3–17.1)	12.8 (9.1–15.7)	0.957	16.2 (15.6–18.5)	17.3 (14.2–33.5)	0.912
CD3^−^/CD16 ^+^ CD56^b^^,^^c^	10.1 (5.0–14.7)	8.4 (3.4–13.4)	0.376	6.4 (5.4–8.8)	6.7 (4.3–9.1)	0.708	7.1 (4.9–7.6)	5.2 (4.9–13.2)	0.971
CD3^+^/CD16 ^b^ ^,^ ^c^	8.8 (4.9–13.9)	6.8 (3.2–12.1)	0.361	5.7 (4.9–8.0)	6.0 (3.6–8.6)	0.789	6.6 (4.5–7.0)	4.9 (4.1–12.2)	0.853
CD3^+^/CD56 ^b^ ^,^ ^c^	6.6 (2.8–11.1)	5.6 (2.2–9.2)	0.405	4.4 (3.8–6.2)	4.3 (2.9–6.2)	0.509	4.4 (2.6–5.1)	4.1 (2.8–9.7)	0.684
CD4^+^/CD8 ^b^ ^,^ ^c^	1.2 (0.9–1.6)	1.3 (0.9–1.8)	0.649	1.2 (1.0–1.4)	1.1 (0.8–1.6)	0.735	0.8 (0.6–1.2)	0.8 (0.6–1.5)	0.684
CD4 ^−^ /CD8 ^−^ ^b^	2.4 (1.8–3.5)	2.6 (2.4–3.4)	0.307	3.0 (2.4–4.3)	3.1 (2.5–3.8)	0.735	3.0 (2.5–3.6)	2.6 (2.2–3.3)	0.393
CD14 ^b^ ^,^ ^c^	7.9 (4.7–9.7)	7.7 (5.9–9.3)	0.668	7.5 (5.9–10.0)	8.7 (6.5–13.4)	0.343	7.9 (7.0–8.6)	9.4 (8.4–14.6)	0.143
CD14^+^/TLR ^b^	93.3 (88.6–94.8)	87.1 (85.1–92.6)	0.025*	91.3 (90.2–92.5)	91.3 (84.8–92.6)	0.580	89.0 (85.4–92.3)	89.5 (83.3–92.3)	0.853

*P*-value of the Mann–Whitney test.

DCC, delayed cord clamping; ICC, immediate cord clamping; MSCs, mesenchymal stem cells; HPCs, hemopoietic progenitor cells; VSELS, very small embryonic-like stem cells; EPCs, endothelial progenitor cells; CRP, c-reactive protein.

* Statistically significant.

a Cell counts are expressed as number of cells per million cytometric events.

b Percentage to total lymphocytes.

c Friedman test suggesting a significant difference between day-one values and day-10 or day-30 values in neonates with DCC.

Neonates with late-onset sepsis had significantly higher median values of early (20 vs. 0.3 cells per million cytometric events, *p* = 0.011) and late EPCs (32 vs. 8 cells per million cytometric events, *p* = 0.033) compared to neonates with no sepsis at birth. Also, neonates with BPD had significantly higher median values of late EPCs (27 vs. 8 cells per million cytometric events, *p* = 0.041) compared to neonates with no BPD at birth (Table 4). Finally, in regression analysis, DCC was significantly positively associated with MSCs (b 0.89, 95% CI 0.13, 1.45, *p* = 0.023) and HPCs (b 0.85, 95% CI 0.11, 1.89, *p* = 0.046), and negatively with VSELs (b −0.93, 95% CI −0.34, −1.67, *p* = 0.049), and late EPCs (b −0.82, 95% CI −0.33, −1.86, *p* = 0.044), adjusted for gestational age and birth weight (Table 5).

**Table 4:** Hematological parameters between neonates with and without LOS and BPD on the first, 10th and the 30th day of life.

Hematological parameters	LOS (*n* = 6)	Non-LOS (*n* = 34)	*p*	BPD (*n* = 6)	Non-BPD (*n* = 34)	*p*
MSCs ^a^	525 (316–2,282)	363 (252–960)	0.644	322 (82–734)	400 (316–10,640	0.343
HPCs ^a^	242 (50–362)	137 (88–217)	0.592	117 (50–217)	142 (96–309)	0.644
VSELs ^a^	60 (24–99)	21 (8–73)	0.118	90 (40–110)	21 (10–70)	0.109
Early EPCs ^a^	20 (1–26)	0.3 (0.08–5)	0.011*	17 (1.9–23)	0.3 (0.01–0.5)	0.082
Late EPCs ^a^	32 (22–44)	8 (0.3–20)	0.033*	27 (22–38)	8 (0.3–20)	0.041*
CD3^+^ ^b^	76 (72–84)	74 (65–80)	0.541	71 (54–82)	74 (69–80)	0.592
CD3^+^/CD4^+^ ^b^	57 (54–66)	51 (46–59)	0.383	52 (38–66)	55 (47–59)	0.956
CD3^+^/CD8^+^ ^b^	20 (14–27)	19 (16–25)	0.926	18 (15–21)	19 (16–25)	0.671
CD19^+^ ^b^	10 (6–17)	11 (8–14)	0.698	9 (8–17)	11 (7–14)	0.541
CD3 ^−^ /CD16 ^+^ CD56^+^ ^b^	11 (6–17)	8 (4–14)	0.425	18 (8–28)	7 (4–14)	0.118
CD3^+^/CD16^+^ ^b^	11 (6–16)	7 (3–13)	0.404	17 (8–26)	6 (3–13)	0.100
CD3^+^/CD56^+^ ^b^	9 (4–12)	5 (2–10)	0.306	14 (7–23)	4 (2–10)	0.071
CD4^+^/8^+^ ^b^	1.1 (1.1–1.8)	1.2 (0.9–1.8)	0.985	1.1 (0.9–1.9)	1.2 (0.9–1.8)	0.868
CD4 ^−^ /CD8 ^−^ ^b^	3 (2–3.9)	2.6 (2.1–3.3)	0.698	2.6 (2.4–3.5)	2.6 (2.0–3.5)	0.839
CD14^+^ ^b^	5.2 (3.3–8.9)	7.9 (5.9–9.7)	0.197	6.4 (3.3–8.9)	7.8 (5.9–9.7)	0.541
CD14^+^/TLR ^b^	92 (91–93)	89 (85–94)	0.566	90 (86–93)	91 (85–94)	0.754

*P*-value of the Mann–Whitney test.

LOS, late-onset sepsis; BPD, bronchopulmonary dysplasia; MSCs, mesenchymal stem cells; HPCs, hemopoietic progenitor cells; VSELS, very small embryonic-like stem cells; EPCs, endothelial progenitor cells.

* Statistically significant.

a Cell counts are expressed as number of cells per million cytometric events.

b Percentage to total lymphocytes.

**Table 5:** Linear regression analysis of the association between DCC and MSCs, HPCs, VSELs, early and late EPCs, adjusted for gestational age and birthweight.

Variables	*b*	95%CI	*p*
MSCs			
DCC	0.89	0.13, 1.45	0.023*
Gestational age	2.08	0.25–3.67	0.018
Birth weight	−0.55	−0.17 to −1.32	0.511
HPCs			
DCC	0.85	0.11, 1.89	0.046*
Gestational age	2.30	0.14–3.98	0.025
Birth weight	−0.96	−0.22 to −1.78	0.332
VSELs			
DCC	−0.93	−0.34, (−1.67)	0.049*
Gestational age	0.20	−0.34 to −1.54	0.843
Birth weight	−0.56	−1.25 to −1.67	0.580
Early EPCs			
DCC	−0.75	0.56, (−1.87)	0.139
Gestational age	1.22	−0.45 to −2.41	0.267
Birth weight	−1.49	−2.66 to −1.08	0.174
Late EPCs			
DCC	−0.82	−0.33, (−1.86)	0.044*
Gestational age	0.14	−1.55 to −2.01	0.901
Birth weight	−0.58	−1.89 to −2.13	0.612

DCC, delayed cord clamping; MSCs, mesenchymal stem cells; HPCs, hemopoietic progenitor cells; VSELS, very small embryonic-like stem cells; EPCs, endothelial progenitor cells; CI, confidence interval.

* Statistically significant.

Moreover, neonates with DCC had significantly higher median values of CD19^+^ (12.8 vs. 10.1 percentage to total lymphocytes, *p* = 0.029) and CD14^+^/TLR (93.3 vs. 87.1 percentage to total lymphocytes, *p* = 0.025) and lower median values of CD3^+^/CD4^+^ (49.6 vs. 59.2 percentage to total lymphocytes, *p* = 0.034), compared to neonates with ICC at birth (Table 3). Also, as expected, neonates with DCC had significantly higher median hemoglobin level compared to neonates with ICC (11.6 vs. 9.7 g/dl, *p* = 0.048), on the 30th day of life.

## Discussion

Our findings are in line with previous evidence suggesting that DCC is associated with enhanced levels of MSCs and HPCs compared to ICC in preterm neonates, even after adjusting for gestational age and birth weight. Also, in neonates with DCC, MSCs and HPCs were significantly reduced from birth within the first month of life. MSC is a significant cell type in the stem cell family with notable biological characteristics, including paracrine secretion of different bioactive factors, low immunogenicity, high plasticity, strong multidirectional differentiation ability, wide source, and low risk of teratogenesis and tumorigenicity (26). MSCs participate in hematopoietic support, nutrient provision, endogenous stem/progenitor cell activation, tissue damage repair, inflammation removal, immunomodulation, neovascularization promotion, chemotaxis and migration, anti-apoptosis, antioxidant, anti-fibrotic, and homing (26). MSCs may exhibit lower functional maturity in preterm newborns, as well as less effective immunomodulation and occasionally decreased differentiation potential (27). HPCs are vital for the development of the immune system and the oxygenation of tissues since they produce all blood and immune cells. While their “naïve” immunological profile helps to lower immune reactivity, HPCs can proliferate quickly and produce a variety of immune cell lineages in term newborns, while preterm neonates may have defective HPC production of mature immune cells (28, 29). According to earlier publications, reduced EPCs and CD34^+^ hematopoietic stem cell in placental residual blood volume indicate that there is a placental reserve volume available to give placental to fetal transfusion using DCC (14). Because of the 1:1 placenta-to-body ratio, the placenta carries about 50% of the blood volume of early preterm neonates. The smallest preterm neonates may lose as much as 50% of their blood volume due to ICC (30). Zhou et al. found that they could drain 19 ml/kg of leftover fetal/placental blood, or almost 25% of the blood volume possibly available for the preterm neonates, even after DCC at 60 s (30). Moreover, Javad et al. (31) showed that MSCs may be extracted from preterm UCB as early as 24 weeks, and that the 32–36 week groups had a considerably higher number of colonies per 100 million mononuclear cells plated than the 37–40 week groups. Notably, the lowest gestational age had the largest percentage of MSCs, indicating that very and extremely preterm births may produce greater MSC percentages (32).

Interestingly, we found lower median values of VSELs and late EPCs in neonates with DCC at birth, compared to neonates with ICC, while DCC was associated with lower levels of VSELs and EPCs, after controlling for gestational age and birth weight. EPCs regulate their microenvironments to promote organ development, homeostasis, and tissue regeneration (33). By targeting tissue damage sites to restore vascular integrity and guarantee proper endothelial function, EPCs play a crucial role in vascular repair and regeneration (34). A population of early-development stem cells known as VSELs is thought to be deposited in bone marrow and other postnatal tissues and organs. When exposed to intense exercise, infection, or tissue/organ damage, their number in peripheral blood rises, and they are enriched in UCB (35–37). The lower levels of VSELS and EPCs in neonates with DCC compared to ICC could be explained by several effects of UBC-derived MSCs. MSCs release antioxidants such as stanniocalcin-1 and anti-inflammatory factors via exosomes, including interleukin-6, interleukin-8, metalloproteinase-9, tumor necrosis factor-alpha, and transforming growth factor-beta (38, 39). According to earlier research, progenitor cell populations dynamics may be significantly impacted by the cytokines released from the placental tissues (40, 41). Consequently, DCC may lead to a decrease in blood progenitor cell levels by increasing the transmission of placental factors, which aid in the homing process to the target organ receptors (15). In our cohort, we observed lower median concentrations of circulating VSELs and late EPCs in the DCC group, likely reflecting a rapid tissue homing or phenotypic modulation of rare progenitors following placental transfusion.

In our study, CD19^+^ and CD14^+^/TLR were also found to be higher, while CD3^+^/CD4^+^ were lower in neonates with DCC, compared to neonates with ICC. According to earlier research, preterm neonates with DCC have greater percentages of CD19^+^ lymphocytes in their cord blood than neonates with ICC (42). Additionally, preterm neonates with DCC had a lower CD3^+^/CD4^+^ percentage on the seventh day of life than those with ICC, even though there was no difference observed in the cord blood (42). Bussel et al. found that the CD19^+^ B-lymphocyte percentages in preterm neonates were higher during the first eight weeks of life than in term neonates (43). Furthermore, a considerably larger production of CD34^+^ cells was seen with older gestational age, which may be related to improved placental transfusion (44, 45). B- and T-lymphocytes are major components of the immune system, and prior research has shown that neonates with culture-confirmed sepsis had lower absolute counts and CD4^+^ T-lymphocyte ratios than those without (46). Most of the information that is currently available about CD14^+^/TLR focuses on its role as a co-receptor that works with TLR4 to promote inflammatory response (47). However, new data indicate that CD14^+^ plays a variety of roles in immune regulation of different cells and tissues, such as detecting phospholipids on the external plasma membrane (48) to clear apoptotic cells and mediating both inflammatory and tolerogenic reactions to changes in the local microenvironment (49).

Although we did not find any difference in late-onset sepsis or BPD rates between neonates with DCC and ICC, neonates without late-onset sepsis had significantly lower median values of early and late EPCs compared to neonates with sepsis, and neonates without BPD had significantly lower median values of late EPCs compared to neonates with BPD. The ability of VSELs to develop into epithelial cells has been shown to be particularly significant in the lungs, because it helps maintain the lungs' proper structure and function (50). According to recent research, the lung's blood vessels actively support healthy alveolar growth (51), and angiogenesis disturbance can hinder alveolarization and potentially be a major factor in the pathophysiology of BPD (52). From bone marrow, EPCs can migrate to the peripheral circulation, where they aid in the development of new blood vessels and the healing of damaged endothelium (53). In a previous study by Borghesi et al., preterm neonates weighing less than 1,500 g or less than 32 weeks of gestational age who later developed BPD had significantly lower ECFCs compared to neonates without BPD (17). A subsequent study of the same group found no association between any of the prenatal or postnatal factors examined and the circulating angiogenic cell counts at birth, seven, or 28 days of life (21). Similarly, Paviotti et al. found no association between neonatal outcomes, such as BPD, and the EPC levels at birth (22). Preterm neonates with or without oxygen dependence at 28 days showed comparable levels of EPC shortly after birth, according to Qi et al. (19), while some EPC subtypes were significantly lower on day seven and 21 in neonates that developed BPD than those without BPD. Lastly, Safranow et al. discovered that neonates with severe retinopathy of prematurity, BPD, and late-onset sepsis had higher levels of cord blood EPCs (20). Overall, these results imply that preterm ECFCs and EPCs, which are more susceptible at this stage of development than term cells, can be greatly impacted by prenatal and postnatal stresses. It is possible, nevertheless, that other cell morphologies could be more associated with the development of BPD (54).

## Limitations

The limitations of our study should be acknowledged. First, our single-center cohort study results might not be generalizable. Second, we acknowledge that the sample size of our cohort was relatively small, although comparable to previous studies. Moreover, we were able to examine the hemopoietic factors of our neonates at three time-points, within the first month of life. Also, as per our institution's practices, DCC was the preferred method of cord clamping, unless contraindicated, when ICC was performed. We acknowledge that, as per our study design, neonates with ICC might have been born more compromised compared to neonates with DCC. Perinatal stress, or any form of oxidative stress (i.e., increased maternal age, maternal diabetes, preeclampsia, fetal-growth restriction) has been found to affect the levels of MSCs and HPCs. Previous evidence suggests that, compared to more differentiated cell types, MSCs are more susceptible to oxidative stress and exhibit lower antioxidant activity (55). According to conflicting evidence, placenta-derived MSCs from women of advanced age had either reduced (56), or similar (57) proliferative and self-renewal capabilities than those from younger women. Evidence is also inconclusive regarding preeclampsia, as some authors found that there were less EPCs isolated from umbilical cord blood in preeclamptic patients than in healthy ones (58), while others showed that preeclamptic women had increased EPC proliferation and unchanged EPC counts (59). Also, low proliferation rates, decreased cell viability, increased cell death, and low mitochondrial activity have all been observed for umbilical cord-derived MSCs from gestational diabetes-affected pregnancies (60). Finally, MSCs obtained from fetal-growth restricted pregnancies exhibit reduced levels of cell proliferation, angiogenesis capacity, and restricted multipotency as compared to healthy placenta (61). Notable, no neonate in our cohort suffered perinatal asphyxia, while APGAR scores (as an indicator of the perinatal status) were similar between neonates with DCC and ICC. Although we recorded no significant differences in any of the perinatal variables examined, we could not exclude a potential bias in the hematopoietic results measured. In addition, as per our study design, we could only examine the percentages of basic immunophenotype of lymphocytes, MSCs, HPCs, VSELs, and EPCs in the umbilical cord of every neonate. The lymphocyte percentage alone does not fully reflect immune competence, and confounding factors (i.e., antenatal steroid administration) may impact the results. However, due to the small sample size, a regression analysis to adjust for those confounding factors would not be feasible. Also, we could not exclude that the elevated EPCs in neonates with sepsis and BPD may suggest reactive or compensatory rather than causative repair mechanism. Finally, we could not examine the association between hematopoietic parameters and certain neonatal outcomes (i.e., retinopathy of prematurity, early-onset sepsis, necrotizing enterocolitis) as their incidence was low in our cohort. Of note, although we found significant differences in EPCs between neonates with and without late-onset sepsis, and with and without BPD, it should be noted that no etiological association could be made given the multifactorial nature of these diseases.

## Conclusion

Our study suggests that, in very preterm neonates, DCC is associated with higher levels of MSCs and HPCs, while lower levels of VSELs and late EPCs, compared to ICC. Significantly higher median values of early and late EPCs were found in neonates with late-onset sepsis compared to those without late-onset sepsis, and significantly higher median values of late EPCs were found in neonates with BPD compared to those without BPD. Our findings indicate that umbilical mesenchymal and progenitor cells may be of significance in the development of neonatal morbidities, including late-onset sepsis and BPD. Future longitudinal studies are warranted to examine the early immune status by assessing the functional assays or cytokine profiling in UCB and evaluate the etiological association between UCB hemopoietic parameters and neonatal outcomes, notwithstanding the association between DCC and long-term neonatal outcomes.

## Data Availability

The raw data supporting the conclusions of this article will be made available by the authors, without undue reservation.
