# Supplementary material for: Impact of delayed cord clamping on mesenchymal, hemopoietic progenitor, and immune cells in very preterm neonates
Source: Front Pediatr. 2025 Nov 25;13:1698512. doi: 10.3389/fped.2025.1698512 (PMC12685807; doi:10.3389/fped.2025.1698512)
Supplement: Supplementary file 1 [file Table1.docx]

**Supplemental Tables**

**Supplemental Table:** Data on monoclonal antibodies used for the study

| **Monoclonal Antibody** | **Clone** | **Manufacturer** |
| --- | --- | --- |
| CD34 PE-CY 7 | 4H11 (APG)MOUSE IgG1 | EXBIO |
| CD34 PE-CY 5 | 581,MOUSE IgG1 | EXBIO |
| CD90 PE-CY 5 | 5E10 , MOUSE IgG1 | EXBIO |
| CD45 PE- DyLight 594 | MEM-28 MOUSE IgG1 | EXBIO |
| CD184/CXCR4- PE | 12G5,MOUSE IgG2a | EXBIO |
| CD73 FITC | AD2,MOUSE IgG1 | EXBIO |
| CD105 PE | MEM 229,MOUSE IgG2a | EXBIO |
| CD133 FITC | EMK08 | INVITROGEN |
| CD3+FITC/CD19+PE | UCHT-1/J3-119 | BECKMAN COULTER |
| CD3+FITC/CD4 +PE | UCHT-1/13B8.2 | BECKMAN COULTER |
| CD3+ FITC/CD8+ PE | UCHT-1/B9.11 | BECKMAN COULTER |
| CD3-FITC/16+56+ PE | UCHT-1/3G8-N901(NKH-1) | BECKMAN COULTER |
| CD14+FITC | MEM-15 MOUSE IgG1 | EXBIO |
| TLR4+ (CD284) PE | HTA125, MOUSE IgG2a | BIOLEGEND |
